# Problematic social media use and negative risk-taking behavior among college students: the chain mediating role of grit and academic procrastination

**DOI:** 10.3389/fpubh.2026.1803931

**Published:** 2026-07-02

**Authors:** Yan Li, Lie Luo, Lehong Zhou, Yifu Qiu

**Affiliations:** 1 School of Marxism, Guangdong University of Petrochemical Technology, Maoming, China; 2 School of Marxism, Shantou University, Shantou, China; 3 School of Education, Guangzhou University, Guangzhou, China; 4 College Student Psychological Development Guidance Center, Guangdong University of Petrochemical Technology, Maoming, China

**Keywords:** academic procrastination, college students, grit, negative risk-taking behavior, problematic social media use

## Abstract

**Background:**

Social media has greatly facilitated college students' daily lives, yet the academic problems and negative behaviors caused by problematic social media use are becoming increasingly prominent. Based on the self-regulation theory, this study examined the association between problematic social media use and negative risk-taking behavior in college students, and explored the potential roles of grit and academic procrastination in this linkage.

**Method:**

A total of 754 Chinese college students were measured by the convenience sampling method, and the data were analyzed via SPSS 27.0.

**Result:**

Research showed that college students' problematic social media use was significantly negatively correlated with grit, and significantly positively correlated with academic procrastination and negative risk-taking behaviors. Academic procrastination acted as an independent mediator between problematic social media use and negative risk-taking behaviors, while grit acted as an independent mediator between problematic social media use and academic procrastination. Grit and academic procrastination played a chain mediation role between problematic social media use and negative risk-taking behaviors.

**Conclusion:**

This study reveals a positive correlation between problematic social media use and negative risk-taking behavior, as well as the potential roles of grit and academic procrastination in this relationship. Public health interventions should focus on the association between problematic social media use and risk-taking behaviors among college students, with particular attention to psychological and behavioral indicators such as grit and academic procrastination, thereby providing theoretical references for academic guidance and risk-taking behavior prevention.

## Introduction

In recent years, with the rapid development of digital technology, online social media has profoundly changed college students' lifestyles (1). On the one hand, the widespread use of social media has made college students' learning and life more convenient. Social media provides abundant learning resources, enables students to update the latest news globally, communicates with family and friends anytime and anywhere, enjoys leisure and entertainment to the fullest, and shares as well as records their lives (2). On the other hand, social media is also flooded with various types of information, mixed in quality and difficult to distinguish between true and false. Excessive use of social media may also have negative effects on the physiological, psychological, and social functions of college students (3–5). An increasing number of studies have found that problematic social media use will lead to academic stress, academic procrastination, and decreased academic performance (6). Frequent web browsing and short video watching can distract college students' attention and weaken their motivation to work toward their goals. Long-term exposure to negative phenomena on social media may also lead to risk-taking behaviors (7). Previous studies have primarily examined the mechanisms underlying problematic social media use among college students from various dimensions, including academic performance (e.g., academic achievement, academic procrastination), interpersonal relationships (e.g., social anxiety, social comparison), and mental health (e.g., depression, subjective wellbeing) (2, 8, 9). However, few studies have explored the association between problematic social media use and negative risk-taking behaviors from the perspective of resource depletion.

The self-regulation theory posits that self-regulation is the ability to modify one's own behavior, enabling individuals to adjust their actions to meet the demands of social and situational contexts (10). Effective self-regulation enables individuals to achieve academic and professional success, maintain harmonious interpersonal relationships, and foster mental health and behavioral adaptability (11). It is important to recognize that self-regulation relies on certain limited resources, which, much like energy, enable individuals to demonstrate persistent willpower when facing challenges or stress (12). When individuals become addicted to social media use and excessively deplete these resources, they enter a state of self-depletion, rendering them incapable of effective self-regulation. This may lead to negative risk-taking behaviors such as drinking, smoking, and skipping classes. Therefore, this study aims to examine the association between problematic social media use and negative risk-taking behaviors, and to explore the potential roles of grit and academic procrastination in this relationship.

### Problematic social media use and negative risk-taking behavior

Problematic social media use refers to an individual's excessive focus on social media, having a strong motivation to use it, and spending a great deal of time and energy on it, which can harm their learning, interpersonal relationships, and mental health (3). Although problematic social media use is not explicitly classified as “behavioral addiction” (non-material related disorder) in the International Classification of Diseases (11th Edition) and the American Diagnostic and Statistical Manual of Disorders (5th Edition), However, an increasing number of researchers believe that it shares similar characteristics with other non-substance addictions, including excessive use, withdrawal symptoms, and functional impairment (13), and therefore consider it a potential behavioral addiction for research (14, 15). Research shows that excessive use of social media has a negative impact on students' academic performance, leading to symptoms such as insomnia, loneliness, anxiety, and depression (16, 17).

College students are at the forefront of social media usage and are the most likely to use it. They are the main group for problematic social media use (18) and also a high-risk group for negative risk-taking behavior. According to Erikson's theory of psychosocial development, college students are in early adulthood and face the task of establishing self-identity and avoiding role confusion. During this period, college students' self-awareness significantly increases, and they begin to engage in more life explorations. Especially in the context of Chinese culture, influenced by exam-oriented education, students have been burdened with heavy academic tasks and intense competition from primary school to high school (19). After entering university, many students tend to relax their mindset and show strong curiosity toward the external environment and new things, actively trying new learning methods and social approaches. However, the cognitive abilities of college students are not yet fully developed, which may lead them to engage in negative risk-taking behaviors during exploration due to various factors and temptations. Research has found that problematic social media use is a risk factor for college students' risk-taking behavior (20). Excessive addiction of college students to social media can easily prone to neglecting their studies, failing to complete existing learning tasks such as course assignments and final papers on time (21), which may ultimately trigger potentially serious negative consequences such as falsifying data, cheating in exams, plagiarism in papers, disinterest in learning, non-suicidal self-harm, etc. (7). Therefore, this study hypothesizes that:

*H1*. Problematic social media use is significantly positively correlated with negative risk-taking behavior.

### The mediating role of academic procrastination

Academic procrastination may be one of the mechanisms linking problematic social media use to negative risk-taking behavior among college students. Academic procrastination is a manifestation of individual self-regulation failure in learning. It is a maladaptive coping style toward learning and is prevalent among college students (22). Severe academic procrastination can lead to a decline in academic performance and trigger negative emotions such as tension, anxiety, and depression (23). In recent years, with the excessive use of social media, college students' academic procrastination behavior has become particularly prominent (22). The rapid development of social media has provided people with richer ways to relax and entertain (1). However, at the same time, improper or excessive use of social media can easily immerse them in the virtual world, leading to academic procrastination and affecting their learning, work, and life (24, 25). Academic procrastination further exacerbates negative emotions such as anxiety, guilt, and self-blame among college students, leading to chaotic time management and the accumulation of problems. Research has found that individuals who frequently procrastinate tasks often experience continuous anxiety due to unfinished or approaching task deadlines, which may lead to risk-taking behaviors (26).

The self-regulation theory holds that individual behavior is driven by beliefs or expectations, and to achieve goals, individuals will monitor, guide, and modify their cognition, emotions, behaviors, and motivations (27). College students use social media to satisfy their immediate emotional needs and information acquisition, but excessive use may lead to the failure of their self-monitoring system, resulting in unconscious short-term pleasure behaviors such as frequent browsing of web pages and short videos (5). This continuously depletes individuals' self-regulating resources, leading to academic procrastination, stress, anxiety, avoidance, and other psychological issues. Further may lead to a decline in impulse control system function, resulting in negative risk-taking behaviors such as skipping classes, cheating, smoking, and alcohol abuse, etc. Based on this, this study hypothesizes that:

*H2*. Academic procrastination plays a mediating role in the relationship between problematic social media use and negative risk-taking behavior.

### The chain mediating role of grit and academic procrastination

On the other hand, there may be variables (e.g., grit) between problematic social media use and academic procrastination. Grit is often regarded as a personality trait, referring to an individual's sustained passion and endurance when facing long-term goals (28). If a person has a strong interest and passion in a certain field, even in the face of distractions or setbacks, they can still maintain the duration and energy needed to achieve long-term goals. Duckworth et al. (28) pointed out that talent or luck may be important for success, and grit is not talent or luck, but equally important, or even more important for success. Excessive use of social media by college students can frequently disrupt their daily learning routine, causing conflicts between the brain's preference for instant gratification (e.g., scrolling through short videos) and long-term goals (e.g., studying), resulting in difficulty concentrating and weakening their ability to maintain long-term focus. And concentration is one of the fundamental elements of grit. Meanwhile, problematic social media use often leads to an imbalance in time allocation, squeezing out time that should be used for learning, work, or personal growth. This shift in behavior patterns may weaken college students' ability to plan and execute long-term goals, thereby damaging their grit. That is to say, excessive use of social media may cause temporary damage to an individual's psychological resources (e.g., grit), lead to failure in self-regulation, and subsequently result in behavioral discontrol (e.g., academic procrastination, risk-taking behaviors). Research has found a significant negative correlation between grit and addiction to online games (29, 30). Grit can predict academic performance (31, 32), and Students with high levels of grit may be less likely to procrastinate (33). Grit is a potential protective factor for substance use and other risk-taking behaviors among the youth (34). Some studies have also found that grit mediates the relationship between college students' social media use and academic performance (8). Additionally, the prediction of certain performance behaviors (e.g., negative risk-taking behavior) by grit may be mediated by other variables or processes (35, 36). Therefore, the study hypothesizes that:

*H3*. Grit plays a mediating role in the relationship between problematic social media use and academic procrastination.

*H4*. Grit and academic procrastination play a chain mediating role in the association between problematic social media use and negative risk-taking behaviors.

**Figure 1:**
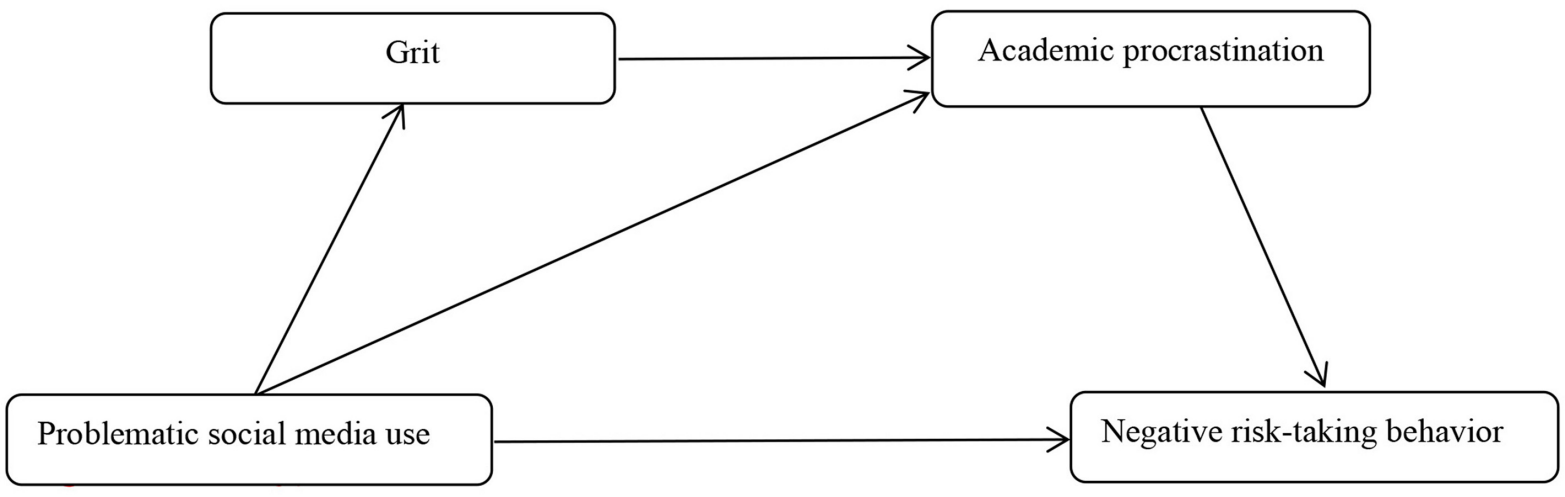
The hypothesized model.

In summary, this study aims to explore the association and internal mechanism between problematic social media use and negative risk-taking behaviors among college students. Considering the relationships among problematic social media use, grit, academic procrastination, and negative risk-taking behaviors, this study proposes the following hypothesized model, as shown in Figure 1.

## Method

### Participants

The participants come from two public undergraduate universities in a large province in southern China. A convenient sampling method was used to distribute anonymous online questionnaires to students in the class, including basic demographic information. After data screening and quality control, 754 valid samples were finally obtained, with an effective rate of 93.3%, including 446 (59.2%) males and 308 (40.8%) females; 448 (59.4%) students in Grade 1, 229 (30.4%) students in Grade 2, and 77 (10.2%) students in Grade 3; 413 (54.8%) students come from rural areas, and 341 (45.2%) students come from urban areas; In terms of monthly household income, 20% of the students monthly household income below CNY 5,000, 43% between CNY 5,000–10,000, 24.9% between CNY 10,000–20,000, and 12.1% above CNY 20,000.

The screening criteria for invalid samples in this study are as follows:

- Answering time < 2 min.
- The answer presents a regular answering pattern, such as a repetition rate of over 80% for the same option.
- Incomplete information filling, such as more than 10% of key information is missing.
- All participants have signed informed consent forms and voluntarily participated in this survey.

### Measures

#### Problematic social media use

The assessment questionnaire of problematic mobile social media usage (PMSMU-AQ), developed by Jiang Yongzhi in 2018 is used to evaluate adolescents' problematic social media use behavior. This scale has a total of 20 items, including five factors which are “increased stickiness”, “physiological damage”, “missed anxiety”, “cognitive failure”, and “guilt”. The scale adopts a Likert 5-point scoring system, where 1 represents “completely disagree” and 5 represents “completely agree”. The main statistical indicators are the total score of the questionnaire and the total score of each factor. The higher the total score of the questionnaire, the more severe the tendency of college students' problematic social media use; The higher the score of each factor, the more severe the tendency toward a certain behavior. Specific items include “I spend a lot of time logging in and checking my moments every day” and “I often feel worried or anxious when my phone suddenly cannot connect to the Internet, and I cannot view social apps”. In this study, the Cronbach's α of the scale was 0.952.

#### Grit

The present study employed the Grit Scale developed by Duckworth et al. (28). It is used to evaluate individuals' sustained effort and enduring passion toward long-term goals. The scale comprises 12 items divided into two domains: consistency of interest and perseverance of effort (six items each). Using a 5-point Likert scale for measurement, ranging from “not at all like me” to “very much like me”. Among these items, items 2, 3, 5, 7, 8, and 11 are reverse-coded. A higher average score indicates higher levels of grit. The Cronbach's α for this scale was 0.692, while the Cronbach's α for consistency of interest and perseverance of effort are 0.744 and 0.803 respectively. Confirmatory factor analysis was used to test the structural validity of the scale, following the two-factor theory structure of the original scale. Residual correlations were only established for semantically similar items within the same dimension based on the modified index. The model fitting results showed that X^2^/df = 4.919, RMSEA = 0.072, GFI = 0.960, NFI = 0.915, CFI = 0.931, TLI = 0.888, SRMR = 0.066. Except that TLI was slightly lower than the ideal critical value, all other fitting indicators reached the statistical acceptable standards, and the SRMR fitting effect was good, indicating that the overall scale structural validity was good and could be used for subsequent empirical analysis.

#### Academic procrastination

The Aitken Procrastination Inventory (API), developed by Professor Aitken in 1982 was used to assess students' academic procrastination behavior. This scale is a single-dimensional self-assessment scale with a total of 19 questions, such as “I always wait until the last minute to start doing things” and “I often work hard to meet deadlines”. Among these items, nine questions (i.e., items 2, 4, 7, 11, 12, 14, 16, 17, and 18) are reverse-coded. Using a 5-point rating, 1 represents “completely disagree” and 5 represents “completely agree”. The higher the score on the scale, the more severe the academic procrastination level of the participants. In this study, Cronbach's α of the scale was 0.807.

#### Negative risk-taking behavior

Referring to the scale on the Adolescent Risk-Taking Questionnaire (ARQ) developed by Gullone et al. (37) and revised by Zhang et al. (58), the scale includes four dimensions: thrill-seeking (five items), recklessness (two items), rebelliousness (six items), and antisocial behavior (four items). Among these, the dimensions of recklessness (e.g., engaging in unsafe sexual behavior), rebellion (e.g., smoking), and antisocial behavior (e.g., mocking and bullying others) are usually regarded as indicators of negative risk-taking behavior, and have been proven to be applicable for measuring negative risk-taking behavior among Chinese adolescents (38). Therefore, this study intends to use the total score of these three dimensions as a measure of negative risk-taking behavior. The higher the score, the higher the level of negative risk-taking behavior. In this study, the Cronbach's α of this scale is 0.757.

### Data Processing

This study used IBM SPSS 27.0 to perform common method bias testing, descriptive statistics, correlation analysis, *t*-tests, and variance analysis on the data. The PROCESS plugin of the SPSS macro program was employed to conduct the mediation effect test.

## Results

### Common method bias test

This study employed self-reporting for measurement. To eliminate the influence of common method bias, the measurement process adopted standardized administration procedures, uniform instructions, anonymous questionnaire filling, and reverse scoring for some questions, etc. Statistically, the Harman single-factor test was used to conduct the common method bias test on the data. The results showed that there was a total of 14 factors with eigenvalues greater than one without rotation, and the explanatory power of the first factor was 20.76%, which was far below the critical standard of 40% (39). To further assist in verifying the results, all items were loaded onto a single latent factor for confirmatory factor analysis (CFA) to verify the model fit. The results revealed that the model fit was poor (χ^2^/df = 6.696, RMSEA = 0.087, CFI = 0.482, TLI = 0.465), indicating that the common method bias in the data of this study was not serious.

### Descriptive statistics and correlation analysis

From the data analysis of the participants (See Table 1), Significant gender differences were observed in college students' problematic social media use (*t* = −3.737, *p* < 0.001) and negative risk-taking behaviors (*t* = 3.381, *p* < 0.001). Females had higher problematic social media use, while males showed more negative risk-taking behaviors; Students from rural areas also had significantly higher problematic social media use than urban ones (*t* = 2.112, *p* < 0.001). No significant differences were found in problematic social media use and negative risk-taking behaviors among college students regarding grade and monthly household income (*p* > 0.05).

**Table 1:** Comparison of mean and standard deviation of problematic social media use and negative risk-taking behavior in terms of demographic characteristics.

Variable	Category	*N* (%)	PSMU		NRTB	
			Mean (SD)	*t/F*	Mean (SD)	*t/F*
Gender	Male	446 (59.2%)	2.868 (0.809)	−3.737^***^	1.358 (0.352)	3.381^***^
	Female	308 (40.8%)	3.081 (0.706)		1.278 (0.265)	
Grade	Grade 1	448 (59.4%)	2.994 (0.791)	1.425	1.347 (0.341)	2.520
	Grade 2	229 (30.4%)	2.909 (0.744)		1.292 (0.265)	
	Grade 3	77 (10.2%)	2.870 (0.769)		1.301 (0.350)	
Homeplace	Rural areas	413 (54.8%)	3.009 (0.726)	2.112^*^	1.335 (0.333)	0.850
	Urban areas	341 (45.2%)	2.900 (0.827)		1.315 (0.306)	
Monthly household income (CNY)	Below 5,000	151 (20%)	3.018 (0.751)	0.666	1.306 (0.440)	0.328
	5,000–10,000	324 (43%)	2.952 (0.761)		1.335 (0.324)	
	10,000–20,000	188 (24.9%)	2.949 (0.816)		1.321 (0.282)	
	Over 20,000	91 (12.1%)	2.874 (0.780)		1.334 (0.358)	

PSMU, problematic social media use; NRTB, negative risk-taking behavior.

^*^p < 0.05, ^***^p < 0.001.

The mean and standard deviation of the variables, as well as the correlation coefficients between variables, are shown in Table 2. The results showed that there were significant pairwise correlations between problematic social media use, grit, academic procrastination, and negative risk-taking behavior. Among them, problematic social media use was significantly positively correlated with academic procrastination (*r* = 0.400, *p* < 0.001) and negative risk-taking behavior (*r* = 0.179, *p* < 0.001), and significantly negatively correlated with grit (*r* = −0.266, *p* < 0.001); Grit had a significant negative correlation with academic procrastination (*r* = −0.528, *p* < 0.001) and negative risk-taking behavior (*r* = −0.123, *p* < 0.001); The positive correlation between academic procrastination and negative risk-taking behavior was significant (*r* = 0.227, *p* < 0.001).

**Table 2:** Descriptive statistics and correlations for the variables.

Variables	M	SD	1	2	3	4
1 PSMU	2.955	0.775	1			
2 Grit	3.086	0.400	−0.266 ^***^	1		
3 AP	2.681	0.466	0.400 ^***^	−0.528^***^	1	
4 NRTB	1.326	0.321	0.179 ^***^	−0.123^***^	0.227^***^	1

AP, academic procrastination; N, 754; M, means; SD, standard deviation.

^***^p < 0.001, Two-tailed.

### Mediation analyses

The mediating role of grit and academic procrastination between problematic social media use and negative risk-taking behavior was examined using Model 6 in the SPSS macro program PROCESS plugin. Taking problematic social media use as an independent variable, grit and academic procrastination as mediating variables, and negative risk-taking behavior as a dependent variable into the equation, while gender and grade were controlled as covariates. Bootstrap was set to repeat sampling 5,000 times, and the results were shown in Table 3. Regression analysis indicates that problematic social media use has a significant negative correlation with grit (β = −0.136, *p* < 0.001); Problematic social media use has a significant positive correlation with academic procrastination (β = 0.175, *p* < 0.001); Grit has a significant negative correlation with academic procrastination (β = −0.533, *p* < 0.001); The regression coefficients of problematic social media use (β = 0.051, *p* < 0.001) and academic procrastination (β = 0.115, *p* < 0.001) on negative risk-taking behavior were significantly positive; The regression coefficient of grit on negative risk-taking behavior was not significant (β = −0.009, *p* > 0.05).

**Table 3:** Testing for the mediation model.

Predictors	Model 1 (Grit)			Model 2 (AP)			Model 3 (NRTB)		
	β	*SE*	*t*	β	*SE*	*t*	β	*SE*	*t*
Gender	−0.024	0.029	−0.821	−0.090	0.028	−3.204^**^	−0.089	0.023	−3.837^***^
Grade	−0.010	0.021	−0.473	−0.009	0.020	−0.452	−0.029	0.017	−1.740
PSMU	−0.136	0.018	−7.401^***^	0.175	0.018	9.499^***^	0.051	0.016	3.195^**^
Grit				−0.533	0.035	−15.064^***^	−0.009	0.033	−0.256
AP							0.115	0.030	3.808^***^
*R^2^*	0.072			0.361			0.082		
*F*	19.376^***^			105.545^***^			13.441^***^		

β, Coefficients; SE, standard error; CI, confidence interval.

^**^p < 0.01, ^***^p < 0.001.

**Figure 2:**
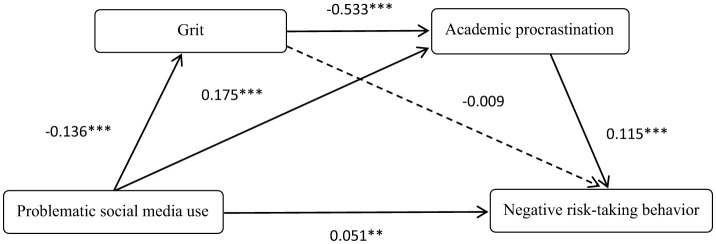
Path coefficients of the mediation model. **p < 0.01, ***p < 0.001.

The results of the statistical mediation effect analysis based on cross-sectional data indicated that after controlling for variables such as gender and grade, the data fitted well with the hypothesized chain mediation model. There was a significant direct association between problematic social media use and negative risk-taking behavior. The direct effect was 0.051, 95% CI [0.020, 0.083]; Problematic social media use was indirectly associated with negative risk-taking behavior through a chain-mediated pathway of grit and academic procrastination, with a total indirect effect of 0.029, 95% CI [0.016, 0.045]. Specifically, the mediating effect was composed of indirect effects generated by two paths: the indirect effect generated by the path of problematic social media use → academic procrastination → negative risk-taking behavior is 0.020, 95%CI [0.009, 0.032]; the indirect effect generated by the path of problematic social media use → grit → academic procrastination → negative risk-taking behavior is 0.008, 95% CI [0.003, 0.014]; Bootstrap 95% CI of both paths did not include 0, indicating that both indirect effects were significant. However, the indirect effect generated by the path of problematic social media use → grit → negative risk-taking behavior was 0.001, and the Bootstrap 95% CI including 0, indicating that the mediating effect of grit between problematic social media use and negative risk-taking behavior was not significant. The results are shown in Table 4 and Figure 2.

**Table 4:** Mediating effects of grit and academic procrastination.

Effects	Estimate	SE	Bootstrap 95%CI	
			Lower	Upper
Total effects	0.081	0.015	0.052	0.110
Direct effect				
PSMU → NRTB	0.051	0.016	0.020	0.083
Indirect effects				
PSMU → Grit → NRTB	0.001	0.005	−0.009	0.011
PSMU → AP → NRTB	0.020	0.006	0.009	0.032
PGI → Grit → AP → NRTB	0.008	0.003	0.003	0.014
Total indirect effects	0.029	0.007	0.016	0.045

PSMU, problematic social media use; AP, academic procrastination; NRTM, negative risk-taking behavior; Bootstrap, 5,000.

## Discussion

The results of the correlation analysis indicated that problematic social media use was significantly positively correlated with academic procrastination and negative risk-taking behavior, while significantly negatively correlated with grit. This suggested that problematic social media use was closely related to the personality traits, academic achievements, and stress coping strategies of college students (40, 41). Further mediation test results indicated that grit and academic procrastination mediate the relationship between problematic social media use and negative risk-taking behavior. The mediation effect is generated by two pathways: one is through the independent effect of academic procrastination, and the other is through the combined effect of grit and academic procrastination.

In this study, problematic social media use, like other risk factors, was found to be correlated with the negative risk-taking behaviors of college students (42, 43). Academic procrastination, as a closely related factor to problematic social media use (44), played a mediating role in the association between problematic social media use and the negative risk-taking behaviors of college students. This result is consistent with previous research on the relationship between problematic social media use and academic performance of college students, as well as research on academic procrastination and risk-taking behavior (42, 43, 45–47). College students are at a critical period of both physical and psychological development, and are highly susceptible to the various influences of social media information, leading to negative and risk-taking behaviors such as smoking, drinking, skipping classes, and unsafe sexual behaviors (48, 49). Compared to other college students, those with a Chinese cultural background mainly use social media for watching short videos, online socializing, and leisure and entertainment. These behaviors are associated with reduced study time and academic procrastination. Academic procrastination often coexists with academic frustration and decreased academic self-efficacy, and is positively correlated with the risk of negative behaviors such as academic violations and impulsive risks among students. According to the self-regulation theory, people attempt to use social media as a means of self-regulation but often fail to succeed and end up in a worse situation instead (50). For example, excessive use of social media consumes self-regulation resources, causing individuals to be in a state of self-depletion for a long time and unable to mobilize effective resources to deal with other important learning tasks, thereby leading to academic procrastination. Academic procrastination will also accumulate learning pressure, resulting in emotions such as self-blame and anxiety (23). To alleviate these negative emotions, college students may choose negative risk-taking behaviors such as cheating, skipping classes, drinking alcohol, staying up late, and smoking as temporary ways to escape from reality. However, this explanation requires further longitudinal research or experimental verification.

It is worth noting that when considering the factor of grit independently, the indirect effect of problematic social media on negative risk-taking behaviors through grit is not significant, but the path of problematic social media use through grit on academic procrastination is significant. Previous studies on grit and negative risk-taking behavior have not reached a consistent conclusion. Some researchers have found that grit is a powerful and significant predictor of academic and life success (28, 44, 51). Adolescents with high levels of grit are significantly less likely to drink alcohol, use marijuana, or fight in the past 30 days, while low levels of grit are associated with drug use and criminal behavior (34). Other researchers believe that grit, as a trait directed toward long-term goals, often achieves its effects through self-regulation processes (52). Academic procrastination is a specific manifestation of self-regulation failure in the academic field (33). Therefore, college students with high levels of grit often have stronger self-regulation abilities and less academic procrastination behavior. The level of grit among college students is negatively correlated with academic procrastination (8). Research has found that grit is a mediating or moderating factor in the impact of internet addiction on procrastination (53). The conclusion of this study supports the indirect role of grit as a mediating factor. The research findings contribute to a better understanding of the association between problematic social media use, grit, academic procrastination, and negative risk-taking behavior.

In addition, the results of this study also found that grit and academic procrastination play a chain-mediated role between problematic social media use and negative risk-taking behavior. Previous studies have shown that individuals addicted to social media often have poor self-regulation abilities (54). Problematic social media use can distract individuals from their long-term goals, weaken their sustained enthusiasm and persistence for long-term goals, reduce their tolerance for boring and long-term tasks, and cause individuals to fail in self-regulation and decline in grit. Research has found that grit is a strong predictor of academic performance (55). Individuals with high grit are better able to resist temptations, manage time, and adhere to study plans, thereby achieving academic success; While those with low grit is significantly positively correlated with academic procrastination (46); That is to say, poblematic social media use is often accompanied by low level of grit, and low level of grit is associated with high level of academic procrastination (53). When this procrastination leads to a backlog of tasks that cannot be completed by the deadline, individuals tend to report tremendous psychological pressure and anxiety. To regulate this negative emotion, individuals may turn to negative risk-taking behaviors (e.g., alcohol abuse, dangerous driving, smoking, skipping classes, cheating, etc.) to vent, escape, or numb themselves (42). However, this study is a cross-sectional design, and the causal chain cannot be confirmed. Future longitudinal studies are needed to verify this. Meanwhile, the research has found that the indirect effect value of this theoretical model was relatively low, indicating that there may be other underlying factors interacting with the relationships between the variables. Numerous studies have shown that the mechanisms driving individual risk-taking behavior are multifaceted, involving various factors such as individual traits, family, peers, and culture (56, 57). Therefore, it is necessary to construct a more comprehensive theoretical framework to study negative risk-taking behavior among college students in the future.

### Limitations and Future Directions

This study has the following limitations: firstly, the sample size is limited to students from two universities, lacking research data on middle school students and other groups. Moreover, the gender and grade distribution of the sample is uneven, resulting in insufficient representativeness of the sample. Future research can further expand the research subjects, increase the sample size of other groups, and generalize the research results to a wider population to verify the relationships between variables; Secondly, the use of self-reported method in this study may lead to measurement errors, which can be overcome in the future by using different measurement tools or methods; Thirdly, this study is based on cross-sectional data and has limitations in conducting causal inference. In the future, specific longitudinal tracking designs or experimental designs can be adopted to further explore the impact mechanism of problematic social media use on negative risk-taking behavior.

## Conclusion

This study aims to investigate the relationship between problematic social media use and negative risk-taking behavior among college students, as well as the mediating role of grit and academic procrastination between the two. The research supports the following hypothesis: first, there is a significant positive correlation between college students' problematic social media use and negative risk-taking behavior; Second, academic procrastination plays a mediating role between problematic social media use and negative risk-taking behavior; Third, grit and academic procrastination play a chain mediating role between problematic social media use and negative risk-taking behavior. The research findings reveal the relationship and internal mechanisms between problematic social media use and negative risk-taking behavior among college students. It suggests that public health intervention measures should focus on the correlation characteristics between problematic social media use and the risk-taking behaviors of college students, and pay attention to psychological and behavioral indicators such as grit and academic procrastination, providing theoretical references for student academic guidance and prevention of risk-taking behaviors.

## Data Availability

The data analyzed in this study is subject to the following licenses/restrictions: the datasets analyzed during the current study are available from the corresponding author on reasonable request. Requests to access these datasets should be directed to psyli2013@gdupt.edu.cn.
