# Correction: Problematic social media use and negative risk-taking behavior among college students: the chain mediating role of grit and academic procrastination

**DOI:** 10.3389/fpubh.2026.1945098

**Published:** 2026-08-03

**Authors:** Frontiers Production Office

**Affiliations:** Frontiers Media SA, Lausanne, Switzerland

**Keywords:** academic procrastination, college students, grit, negative risk-taking behavior, problematic social media use

There was a mistake in Figure 1 as published. The figure was incomplete, as the information in the top text boxes was missing. The corrected figure appears below.

**Figure 1:**
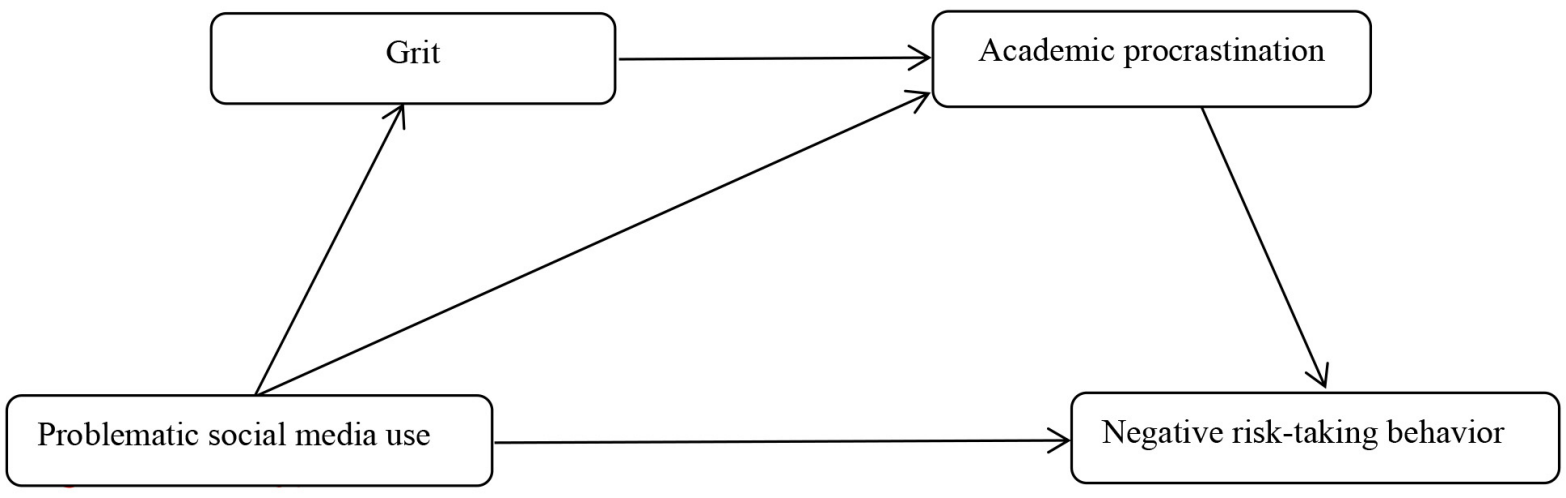
The hypothesized model.

The original version of this article has been updated.

## Generative AI statement

Any alternative text (alt text) provided alongside figures in this article has been generated by Frontiers with the support of artificial intelligence and reasonable efforts have been made to ensure accuracy, including review by the authors wherever possible. If you identify any issues, please contact us.

